# In vitro compatibility of whole blood with commonly used medications in the pediatric trauma setting

**DOI:** 10.1016/j.htct.2026.106518

**Published:** 2026-08-06

**Authors:** Summer Byerley, David Nash, Pamela D Reiter

**Affiliations:** Department of Pharmacy, Children’s Hospital Colorado, 4090 Briargate Parkway Colorado Springs, Aurora, CO, 80920, USA

**Keywords:** Whole blood, Medication, Compatibility

## Abstract

**Introduction:**

Current guidelines do not endorse co-infusion of blood with medication due to concerns for hemolysis and/or clotting. However, concomitant delivery may be required in emergency situations with limited intravenous access. The purpose of this study was to explore the physical compatibility of nine medications used in pediatric emergencies with leukoreduced group O whole blood.

**Methods:**

Blood was manually mixed with study medication and centrifuged at 6500 RPMs for three minutes. A mixture of normal saline and whole blood served as the control. The supernatant was tested for hemolysis using lactate dehydrogenase activity. Each sample was inspected for coalescence or separation. Two separate experiments were performed to demonstrate reproducibility. Compatibility was defined as the absence of gross clot formation/separation provided that lactate dehydrogenase concentrations remained below control values and increased by no more than10% from baseline.

**Results:**

Lactate dehydrogenase activity was measured at two intervals: 15 to 30min and 30 to 60min post-mixing. Cefazolin, epinephrine, succinylcholine, and tranexamic acid in combination with whole blood yielded lactate dehydrogenase concentrations that were stable and below the control values – thus, these were considered compatible at 30–60min. Etomidate and rocuronium could not be analyzed due to either mechanical interference or high analyte concentrations and were considered incompatible. Norepinephrine, calcium chloride, and sodium bicarbonate were considered incompatible as they exceeded the 10% threshold, exhibited lactate dehydrogenase values above control, or showed evidence of separation.

**Conclusion:**

Co-infusion of select medications with whole blood may be considered in the setting of limited intravenous access.

## Introduction

Rapid delivery of blood, along with life-saving medications, is paramount to the survival of a child suffering from acute trauma or hemorrhagic shock. Historically, the co-administration of medications with blood has been discouraged due to the lack of data confirming compatibility. In fact, typical blood bank guidelines promote normal saline (0.9% NaCl) as the only intravenous solution to co-infuse with blood. However, during a trauma event, it is highly likely that concomitant delivery of blood and medication will be required. In this scenario, the provider may be forced to prioritize the provision of either drug therapy or blood and then consider the exploration of supplementary intravenous access sites. Regardless of the choice, care can be delayed.

Compatibility of medications with blood components (e.g., packed red blood cells, fresh frozen plasma, and platelets) has been challenged by the presence of multiple additives (such as citrate-phosphate-dextrose) and nutrient solutions (such as Adsol or Optisol (AS-5) that contain adenine, glucose, saline, and mannitol). These additives are necessary to maintain product stability, prolong shelf life, and avoid clotting. Group-O whole blood, however, contains fewer additives because nutrients within the plasma are maintained. Recently, the use of whole blood demonstrated faster resolution of shock and coagulopathy, lower volumes of transfused blood products, and a reduction in mortality rates among children suffering from acute trauma [1]. The incorporation of whole blood into the care of children with life threatening hemorrhage is now gaining traction and more than ten pediatric centers, including ours, use whole blood as part of their resuscitation strategy [1]. This new approach to hemorrhage resuscitation has ignited interest in the possibility of an infusion of whole blood with select medications simultaneously.

A primary concern surrounding co-administration of blood with medications is the possibility of cell hemolysis and agglutination with resultant spillage of intracellular contents and clotting. The current study aims to assess the in vitro compatibility of leukoreduced group O whole blood with common medications used in a pediatric trauma event. Results from this study will be helpful for future studies evaluating the safety of concomitant drug therapy and whole blood in the setting of limited intravascular access.

## Methods

One unit (500 mL) of chilled (1–6 °C) leukoreduced group O whole blood, that underwent standard laboratory and blood bank processing, was obtained from our local blood bank. The duration of refrigerated storage was 21 days. The unit of blood was preserved with 70 mL of citrate-phosphate-dextrose per the blood bank guidelines. Nine medications commonly used in pediatric emergencies were selected to be mixed with the whole blood (Table 1). Preparation of all medications was performed within the hospital's pharmacy cleanroom using an aseptic technique in a laminar flow hood. The concentrations of medications studied were either (1) commercially available (ready-to-use) from the drug manufacturer or (2) standard diluted concentrations based on institutional guidelines. After preparation and transfer of medications into sterile syringes, samples were transported to the blood bank for further mixing with whole blood and analysis. The volume of medication and whole blood to be tested was based on the calculated volume contained within a typical tubing set at the y-site port for the patient. This volume, determined to be 2 mL, would represent the amount of direct mixing of blood and medication before entering the vessel of a pediatric patient. Therefore, 2 mL aliquots of the leukoreduced whole blood were manually mixed with 2 mL of each medication in individual plastic testing tubes. Moreover, 2 mL normal saline (0.9% NaCl) was added to 2 mL of whole blood to serve as the control sample. Each sample was centrifuged at 6500 RPMs for three minutes per standard laboratory technique, and the supernatant was tested for hemolysis at various times following mixing. Lactate dehydrogenase (LDH) activity was selected to determine hemolysis. This enzyme is found abundantly in red blood cells and is elevated in the presence of cellular destruction. LDH activity was measured for all samples and compared to the control. Concentrations were quantified by reflectance spectrophotometry using the Vitros 7600 LDHI assay (Ortho Clinical Diagnostics, Inc., Rochester, NY, USA). The lower limit of detection of this assay is 41 U/L and the upper limit of quantification is 1000 U/L. The cumulative average coefficient of variability is 2.3 percent. Two separate experiments were performed to demonstrate reproducibility. Baseline LDH was analyzed approximately 15–30 min after mixing (T1) and then again 30–60 min after mixing (T2). All samples were visually inspected for the presence of coalescence or separation. An increase in LDH activity (from T1 to T2) of >10% was predefined as a clinically meaningful change and a signal for incompatibility. This threshold is similar to previous work using LDH as a marker of hemolysis [2]. Percent change in LDH activity was calculated using the following equation: (LDH activity at T2 - LDH activity at T1 / LDH activity at T1) x 100.

**Table 1:** Medications selected to be mixed with whole blood and analyzed for hemolysis.

Medication	Concentration studied
Calcium chloride	100 mg/mL ^1^
Cefazolin	20 mg/mL ^2^
Epinephrine	100 mcg/mL ^2^
Etomidate	2 mg/mL ^1^
Norepinephrine	32 mcg/mL ^2^
Rocuronium	10 mg/mL ^1^
Sodium bicarbonate	1 mEq/mL ^1^
Succinylcholine	20 mg/mL ^1^
Tranexamic acid	100 mg/mL ^1^

1 = commercially available; 2= standard diluted concentration based on institutional guideline.

## Results

During the first experiment, LDH activity was measured at 13 min 45 s after mixing (considered baseline at T1) and again at 29 min 30 s after mixing (considered follow-up at T2). During the second experiment, LDH activity was measured at 38 min 30 s after mixing (considered baseline at T1) and again at 58 min 30 s after mixing (considered follow-up at T2). Experimental results are summarized in Table 2. Two samples (etomidate and rocuronium) could not be analyzed due to an ‘error reading’ on the Vitros 7600 analyzer. Four medications (cefazolin, epinephrine, succinylcholine, and tranexamic acid) had LDH activity below that of the control sample during both experiments and had no gross evidence of coalescence or separation after mixing. However, in the second experiment, succinylcholine and tranexamic both had LDH activity that increased overtime and exceeded the 10% limit. Calcium chloride had LDH activity well above that of the saline control, and this activity increased substantially over time along with evidence of separation after mixing. This was consistent in both experiments. Sodium bicarbonate exhibited unique findings in that the LDH activity remained below control, yet the samples separated after mixing. Finally, norepinephrine had LDH activity below that of control, yet the increase over time approximated 30%. All data supporting the findings of this study are available within the article.

**Table 2:** Lactate dehydrogenase (LDH) activity (U/L) and gross visual inspection of samples after mixing specific medications with whole blood.

Experiment #1 Medication	Baseline LDH U/L (13 min 45 *sec*; T1)	Follow-up LDH U/L (29 min 30 *sec*; T2)	Percent change from T1 to T2	Inspection of mixture*
Control (0.9% NaCl)	93.33	97.63	4.6	1
Calcium Chloride	130.36	160.54	23	2
Cefazolin	68.89	73.69	6.9	1
Epinephrine	72.4	78.37	8.2	1
Etomidate	Could not analyze	Could not analyze	NA	3
Norepinephrine	58.46	75.3	28.8	1
Rocuronium	Could not analyze	Could not analyze	NA	3
Sodium Bicarbonate	67.9	67.97	0.1	2
Succinylcholine	75.21	75.35	0.19	1
Tranexamic Acid	68.84	69.99	1.67	1

Experiment #2 Medication	Baseline LDH U/L (38 min 30 *sec*; T1)	Follow-up LDH U/L (58 min 30 *sec*; T2)	Percent change from T1 to T2	Inspection of mixture*

Control (0.9% NaCl)	89.14	93.06	4.39	1
Calcium Chloride	134.47	183.82	36.7	2
Cefazolin	69.62	77.7	11.6	1
Epinephrine	45.39	65.01	45.5	1
Etomidate	Could not analyze	Could not analyze	NA	3
Norepinephrine	52.36	67.95	29.7	1
Rocuronium	Could not analyze	Could not analyze	NA	3
Sodium Bicarbonate	74.61	76.05	1.9	2
Succinylcholine	80.04	91.51	14.3	1
Tranexamic Acid	75.62	86.37	14.2	1

⁎ Inspection of mixture: 1 = Clear and easily remixed with manual sample inversion; 2 = Sample remained separated despite multiple attempts of sample inversion; 3 = Not analyzed.

## Discussion

This in vitro study examined the compatibility between leukoreduced group O whole blood and various medications used in routine pediatric trauma resuscitation, with findings that have implications for clinical practice. Two experiments involving nine medications were conducted to evaluate cell hemolysis following mixture with whole blood, using changes in LDH concentration over time as a marker. It was not possible to analyze etomidate and rocuronium due to either mechanical interference or high analyte concentrations. Although further dilution of these samples might have yielded a quantifiable LDH concentration, such a step would not reflect clinical practice; consequently, these medications were classified as incompatible. Cefazolin and epinephrine, combined with whole blood, yielded an LDH concentration that remained below that of the saline control and remained free of gross coalescence or separation. In addition, the concentration of LDH over time did not traverse the 10% threshold and thus these two medications can be considered compatible (up to 60 min). Succinylcholine and tranexamic acid combined with whole blood also yielded LDH activity below that of the saline control yet with prolonged exposure, the LDH activity increased by >10%, thereby suggesting that perhaps the combination of these two medications with whole blood should be limited to 30 min or less. Calcium chloride combined with whole blood demonstrated unacceptable levels of LDH activity and had evidence of separation after mixing – thus, calcium chloride was considered incompatible. Although the combination of sodium bicarbonate with whole blood resulted in low LDH activity, the samples consistently exhibited gross separation and were therefore classified as incompatible. Lastly, norepinephrine combined with whole blood resulted in low LDH activity yet the change in LDH activity was nearly three times higher than our pre-determined threshold of 10% and therefore, this medication was classified as incompatible.

Previous research examining the compatibility of medication with blood has been published [[3], [4], [5], [6], [7], [8]]. These earlier works, starting in the late 1970s, used a variety of methods to determine compatibility, including filter clot formation of the blood/medication mixture and hemolysis/agglutination tests (using LDH, potassium, red blood cell indices and/or microscopic and macroscopic examination). Earlier studies in this field primarily consisted of packed red blood cell experiments evaluating opioids and antimicrobials [5]. Some investigations, however, focused on the coadministration of lactated ringers (LR) with whole blood or packed red blood cells. One of the earliest and most frequently referenced experiments was performed by Ryden and Oberman [7]. These authors found that mixing blood with LR resulted in fibrin clot formation under conditions of slow infusion rates, warm ambient temperatures, and a 1:5 blood-to-LR ratio. More recent studies using higher infusion rates and more diluted ratios of blood to LR demonstrated no increase in clot formation when compared to normal saline with blood mixtures [4,8]. These earlier investigations highlight that differences in methodology can alter the outcomes observed. In the current investigation, medications known to be in high demand during an acute trauma resuscitation event were selected and applied under experimental conditions that would mimic ‘real-life’ conditions.

There are limitations to the current work that must be acknowledged. First, institution-specific concentrations were used when the medication required dilution prior to administration. Therefore, external validity and extrapolation of these results to other institutions that use different concentrations is constrained. Second, due to the time required to properly prepare each mixture for testing in the Vitros 7600 analyzer, it was impossible to test for immediate hemolysis (e.g., <5 min) after combining selected medications with whole blood. However, our presumption is that hemolysis risk is related to exposure time and thus shorter exposure time would have yielded a lower risk of hemolysis.

Despite the aforementioned limitations, we believe the results from the current investigation will add to the small body of literature surrounding the coadministration of medications with whole blood. As the use of whole blood becomes more common during trauma resuscitation events, this compatibility information could augment care, especially in the setting of limited intravenous access. Certainly, additional research is warranted to validate the current observation, and more work with different medications would be useful for improving clinical practice.

## Author contributions

S. Byerley was responsible for managing data, conducting the research and creating the first draft. D Nash was responsible for providing oversight and securing resources and materials. PD Reiter was responsible for applying analysis, aiding in method analysis, verifying results, and preparing final manuscript and tables.

## Declaration of generative AI and AI-assisted technologies in the writing process

During the preparation of this work, the author(s) did not use any AI-assisted technology.

## Funding

No outside sources of funding were used to complete this work. This research did not receive any specific grant from funding agencies in the public, commercial, or not-for-profit sectors.

## Conflicts of interest

None of the authors have any conflicts of interest or financial disclosures.

## Data Availability

All data supporting the findings of this study are available within the article.
